# Correction: Cholesterol promotes autoimmune pathology through T follicular helper cells

**DOI:** 10.1038/s41423-025-01350-2

**Published:** 2025-10-03

**Authors:** Wei Li, George C. Tsokos

**Affiliations:** Department of Medicine, Beth Isarael Deaconess Medical Center, Harvard Medical School, Boston, MA USA

**Keywords:** Autoimmunity, Follicular T-helper cells

Correction to: *Cellular & Molecular Immunology* 10.1038/s41423-025-01319-1, published online 10 July 2025

The article “Cholesterol promotes autoimmune pathology through T follicular helper cells”, written by Wei Li & George C. Tsokos, was originally published open access under a Creative Commons Attribution 4.0 International License.

As a result of the authors’ subsequent decision not to publish this article under the open access model, the copyright notice of the article was changed on 12 July 2025 to ©The Author(s), under exclusive licence to CSI and USTC 2025 with all rights reserved.

